# Fermentation induced changes in volatile components of African oil bean (*Pentaclethra macrophylla Benth*) seeds

**DOI:** 10.1002/fsn3.481

**Published:** 2017-05-15

**Authors:** Reginald C. Ohiri, Essien E. Bassey

**Affiliations:** ^1^ Department of Biochemistry Faculty of science University of Port Harcourt Port Harcourt Rivers State Nigeria

**Keywords:** fermentation, Inoculation, Nutritive properties, Therapeutic properties, Volatile components

## Abstract

Fermented African oil bean (*Pentaclethra macrophylla Benth*) seed also referred to as “Ugba” is a known delicacy, mainly consumed by Africans. Human migration has also led to the introduction of this delicacy into most European and American countries. This study shows the changes in volatile components of African oil bean (*P. macrophylla Benth*) seed at different stages of fermentation. A quantity of 0.3 kg each of dehulled and sliced raw sample and cooked unfermented sample were separately ground, while 2 and 4 days fermented samples obtained by inoculating 0.6 kg of cooked, sliced and washed sample with 0.5 g of 4 days fermented seed was divided into two of 0.3 kg each and ground at the second and fourth day of fermentation. GC‐MS analyses of the volatile components showed 9,12‐Octadecadienoic acid (Z,Z)‐ and its esters as highest in raw seed, with total percentage concentration of 96.301, while 9‐Octadecenoic acid, methyl ester, (E)‐ was highest in cooked unfermented seed, with percentage concentration 55.204. Phenol, 2‐methoxy‐3‐(2‐propenyl)‐ and its esters were the highest observed in cooked 2 days fermented seed, with total percentage concentration of 50.596, while 9‐Octadecenoic acid (Z)‐, methyl ester was highest in cooked 4 days fermented seed with percentage concentration of 67.788. Aside from softening the delicacy, a 4 days chance fermentation of cooked *P. macrophylla Benth* seed also reduces the eight component lipids present in the cooked unfermented seed to a more nutriceutical three component lipids (Hexadecanoic acid methyl ester, 9‐Octadecenoic acid (Z)‐methyl ester and Methyl stearate).

## 1 INTRODUCTION

Africans and other locals from developing countries usually practice a traditional fermentation method which involves a spontaneous development of different lactic acid producing bacteria. However, the unavailability of lactic acid bacteria starter cultures for small‐scale processing of local African foods necessitate the adoption of the old traditional method of using a portion of a fermented food product to start a new fermentation batch. This traditional method of microbial seeding also referred to as chance inoculation, in principle resembles the modern starter culture method. In chance inoculation, the initial microbial consortia of the starter raw materials influence the microbial consortia of the fermented product. Fermented African oil bean (*P. macrophylla Benth*) seeds also referred to as “Ugba” is a known delicacy that is mainly consumed in the southern part of Nigeria especially by the Ibos and other ethnic groups. Human migration has also led to the introduction of this delicacy into most European and American countries. Though oil bean seed is mainly composed of proteins (42%), lipids (43%) and carbohydrates (15%) (Njoku & Okemadu, 1989; Ogueke & Aririatu, 2004), studies on the microbiology of its fermentation identified *Bacillus* spp *(Bacillus subtilis*) as the main fermenting organisms (Odunfa & Oyewole, 1986). Other species such *Bacillus pumilus, Bacillus megaterium, Bacillus lichenformis* have also been identified in the fermentation process (Diawara, Sawadogo, Amoa Awua, & Jaobsen, 1998; Odunfa & Oyewole, 1986). The improved taste and palatability of this delicacy after 3 to 4 days of fermentation indicates that the process is not only of lactic acid fermentation but also reduces the anti‐nutrients, enhances the aroma and possibly alters the lipid composition of the product. The aim of this work is to determine the volatile components that are present in fermented African oil bean seed (Ugba) at different stages/days of fermentation.

## 2 EXPERIMENTAL

### 2.1 Sample collection and preparation

Fresh seeds of *P. macrophylla Benth* were purchased from Choba market in Obio‐Akpor Local Government area of River State, Nigeria. A quantity of 0.3 kg of raw uncooked sample was obtained by peeling and slicing 0.5 kg of the raw seeds with a sterilized knife; while a quantity of 2.0 kg of the seed samples were boiled at 100^°^C for 6 hr. The boiled samples were allowed to cool at room temperature, dehulled and sliced using a sterilized knife. A quantity of 0.3 kg each of raw uncooked sample and the cooked unfermented sample were separately ground into a fine smooth paste using Thomas Scientific, (Model 4) Wiley's mill, while the 2 and 4 days fermented samples were obtained by inoculating 0.6 kg of cooked, sliced and washed sample with 0.5 g of a 4 days fermented *P. macrophylla Benth* seed. At the second day of fermentation, a quantity of 0.3 kg of the fermenting sample was ground and the volatile oil was extracted and analyzed to give a 2 days fermented sample, while the remaining 0.3 kg was ground at the 4th day and the volatile oil was extracted and analyzed to give a 4 days fermented sample.

### 2.2 GC‐MS analysis of *P. macrophylla Benth* oil

A hundred gram each of the ground samples of *P. macrophylla Benth* seed paste was added to 3 dm^3^ of distilled water. The oil obtained by hydro‐distillation was collected into hexane and the solution was concentrated by evaporation at room temperature. The oil was analyzed using a combined gas chromatograph model HP 6890 and mass spectrometer model 5973 (AgilentTech.) fitted with a capillary column HP‐5 MS (5% phenylmethylsiloxane) 30.0 m × 250 μm × 0.25 μm, using Helium as a carrier gas at initial column temperature 120°C for 5 min. Thereafter, the column temperature was increased at 5°C per min to 320°C and held for 5 min. Electron impact ionization for mass spectroscopy was done at ionization energy of 70 eV. The oil was diluted with 98% hexane and 2 μl of the diluted sample was automatically injected into AgilentTech model 5973 mass spectrometer. The constituent compounds were identified using the Chem‐Office software attached to the MS library. The names molecular formula and molecular weights of the component oils were ascertained using the database of National Institute of Standard and Technology (NIST).

## 3 RESULTS

**Figure 1:**
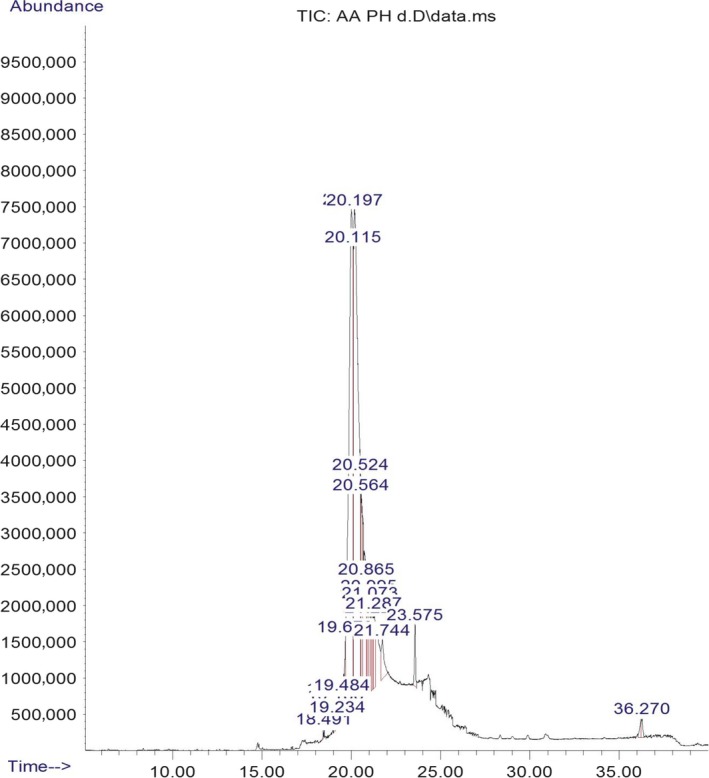
Chromatogram of volatile components of raw *P. macrophylla Benth* seed

**Figure 2:**
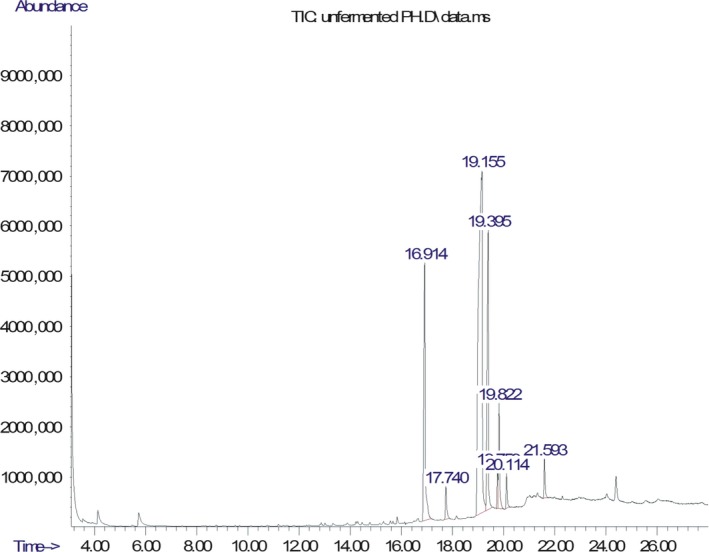
Chromatogram of volatile components of cooked unfermented *P. macrophylla Benth* seed

**Figure 3:**
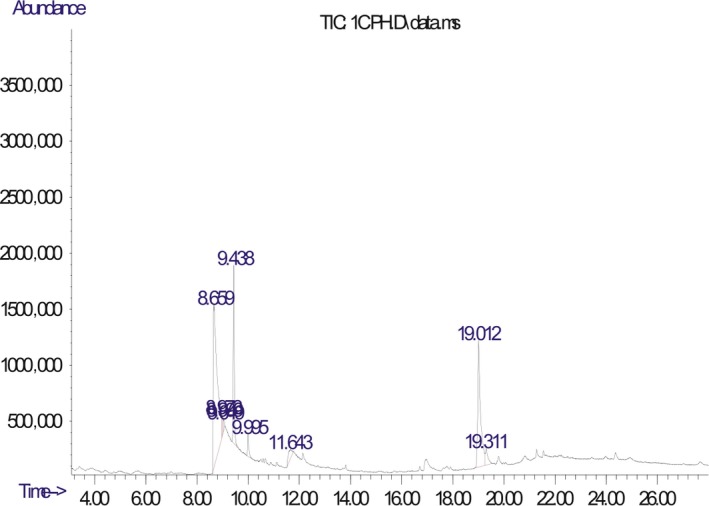
Chromatogram of volatile components of cooked 2 days fermented *P. macrophylla Benth* seed

**Figure 4:**
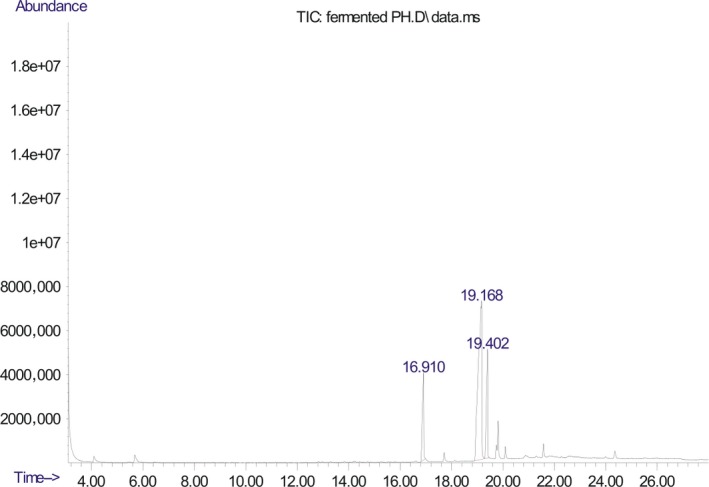
Chromatogram of volatile components of cooked 4 days fermented *P. macrophylla Benth* seed

The chromatogram of the volatile components of raw, cooked unfermented, 2 days fermented and 4 days fermented *P. macrophylla Benth* seed are shown in Figures 1, 2, 3, 4. The highest peaks in the chromatogram of the raw seed were observed at 20.197 min and 20.009 min. (Figure 1), while the highest peaks in the chromatogram of the cooked unfermented seed were observed at 19.155 min and 19.395 min. (Figure 2). Highest peaks in the chromatogram of 2 days fermented seed were observed at 9.438 min and 8.659 min (Figure 3), while 19.168 min and 19.402 min had the highest peaks in the chromatogram of 4 days fermented seed (Figure 4).

The names, retention times, percentage concentration, molecular formula, molecular weight, spectra and structures of the volatile components of raw, cooked unfermented, 2 days fermented and 4 days fermented *P. macrophylla Benth* seed are presented in Tables 1, 2, 3, 4. 9,12‐Octadecadienoic acid (Z,Z)‐ and its esters were the highest volatile component observed in raw *P. macrophylla Benth* seed, with a total percentage concentration of 96.301 and a mean retention time of 20.103 min, while 9‐Octadecenoic acid, methyl ester, (E)‐ was the highest observed in the cooked unfermented seed, with a percentage concentration 55.204 and a retention time of 19.155 min. Phenol, 2‐methoxy‐3‐(2‐propenyl)‐ and its esters were the highest volatile component observed in the cooked 2 days fermented seed, with a total percentage concentration of 50.596 and a mean retention time of 8.920 min., while 9‐Octadecenoic acid (Z)‐, methyl ester was highest in the cooked 4 days fermented seed with a percentage concentration of 67.788 and a retention time of 19.168.

**Table 1:** Results of the analysis by GC‐MS of raw *P. macrophylla Benth* seed

S/N	Compound	Retention Time (min.)	Percentage of the total	Molecular formula	Molecular weight	Structure
1	9,12‐Octadecadienoic acid, (E,E)‐ methyl ester	18.434	0.054	C_19_H_34_O_2_	294.4721	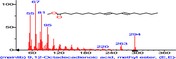
2	11‐Octadecenoic acid, methyl ester	18.491	0.057	C_19_H_36_O_2_	296.4879	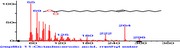
3	Linoleic acid ethyl ester	19.078	0.331	C_20_H_36_O_2_	308.4986	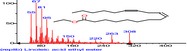
4	n‐Propyl 9‐octadecenoate	19.133	0.256	C_21_H_40_O_2_	324.5450	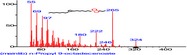
5	n‐Hexadecanoic acid	19.234	0.185	C_16_H_32_O_2_	256.4241	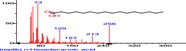
6	9,12‐Octadecadienoic acid (Z,Z)‐	20.103	96.301	C_18_H_32_O_2_	280.4455	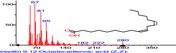
7	i‐Propyl 9‐octadecenoate	19.410	0.471	C_21_H_40_O_2_	324.5411	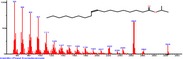
8	9,17‐Octadecadienal, (Z)‐	19.484	0.259	C_18_H_32_O	264.4461	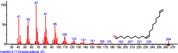
9	1,2Benzenedicarboxylic acid, mono (2‐ethylhexyl) ester	23.575	1.190	C_16_H_22_O_4_	278.3435	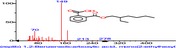
10	Hexane, 2,5‐dimethyl‐	36.208	0.388	C_8_H_18_	114.2285	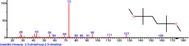
11	trans‐4‐Oxo‐2‐pentenoic acid	36.270	0.507	C_6_H_8_O_3_	128.1259	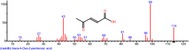

**Table 2:** Results of the analysis by GC‐MS of cooked unfermented *Pentaclethra macrophylla Benth* seed

S/N	Compound	Retention time (min.)	Percentage of the total	Molecular formula	Molecular weight	Structure
1	Hexadecanoic acid, methyl ester	16.914	16.544	C_17_H_34_O_2_	270.4507	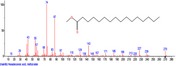
2	Hexadecanoic acid, ethyl ester	17.740	1.763	C_18_H_36_O_2_	284.4772	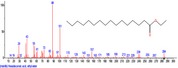
3	9‐Octadecenoic acid, methyl ester, (E)‐	19.155	55.204	C_19_H_36_O_2_	296.4879	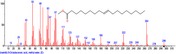
4	Methyl stearate	19.395	16.727	C_19_H_38_O_2_	298.5040	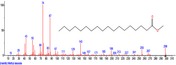
5	Linoleic acid ethyl ester	19.759	1.428	C_20_H_36_O_2_	308.4986	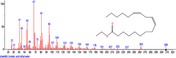
6	(E)‐9‐Octadecenoic acid ethyl ester	19.822	4.933	C_20_H_38_O_2_	310.5145	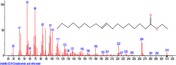
7	Octadecanoic acid, ethyl ester	20.114	1.585	C_20_H_40_O_2_	312.5304	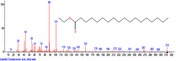
8	Methyl 18‐methylnonadecanoate	21.593	1.817	C_21_H_42_O_2_	326.5570	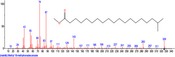

**Table 3:** Results of the Analysis by GC‐MS of cooked 2 days fermented *Pentaclethra macrophylla Benth*

S/N	Compound	Retention time (min.)	Percentage of the total	Molecular formula	Molecular weight	Structure
1	Phenol, 2‐methoxy‐3‐(2‐propenyl)‐	8.920	50.596	C_10_H_12_O_2_	164.2011	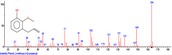
2	Caryophyllene	9.438	15.318	C_15_H_24_	204.3600	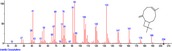
3	Humulene	9.995	2.108	C_15_H_24_	204.3600	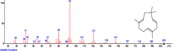
4	Phenol, 2methoxy‐4‐(2‐propenyl)‐,acetate	11.643	2.589	C_12_H_14_O_3_	206.2378	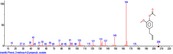
5	9‐Octadecenoic acid, methyl ester	19.012	26.093	C_19_H_36_O_2_	296.4879	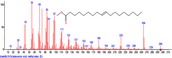
6	Methyl stearate	19.311	3.297	C_19_H_38_O_2_	298.5110	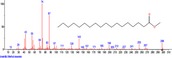

**Table 4:** Results of the Analysis by GC‐MS of cooked 4 days fermented *Pentaclethra macrophylla Benth* seed

S/N	Compound	Retention time (min.)	Percentage of the total	Molecular formula	Molecular weight	Structure
1	Hexadecanoic acid, methyl ester	16.910	14.145	C_17_H_34_O_2_	270.4507	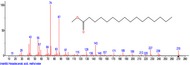
2	9‐Octadecenoic acid (Z)‐, methyl ester	19.168	67.788	C_19_H_36_O_2_	296.4879	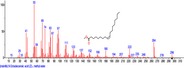
3	Methyl stearate	19.402	18.067	C_19_H_38_O_2_	298.504	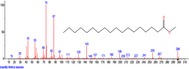

## 4 DISCUSSION

The result of this study shows a progressive decrease in both the number and structural complicity of the volatile components of fermented *P. macrophylla Benth* seed. The high percentage concentration of 9,12‐Octadecadienoic acid (Z,Z)‐ and its esters in the raw seed reveals the hidden nutritional potential of *P. macrophylla Benth* seed. As an essential polyunsaturated omega‐6 fatty acid, 9,12‐Octadecadienoic acid (Z,Z)‐ is required for a proper health as its deficiency has been reported to cause scaling of skin, hair loss (Cunnane & Anderson, 1997), and poor wound healing in rats (Ruthig & Meckling‐Gill, 1999). Irrespective of the high concentration and therapeutic benefit of this dominant fatty acid, the raw *P. macrophylla Benth* seed cannot be consumed because of its unpalatable taste caused by the presence of anti‐nutrients. The presence of cyclic compounds (Phenol,2‐methoxy‐3‐(2‐propenyl)‐, Caryophyllene, Humulene, Phenol,2methoxy‐4‐(2‐propenyl)‐acetate) in 2 days fermented *P. macrophylla Benth* seed shows the ability of the fermenting organisms to induce an enzymatic biochemical process that entails an unstable conversion pathway which generates cyclic structures that may be responsible for the production of aroma. Though these cyclic compounds may have been synthesized as secondary metabolites with different therapeutic properties, the consumption of the cooked unfermented and 2 days fermented seed are been discouraged by their texture, taste and odor. The absence of the cyclic compounds in the 4 days fermented seed may be an indication of the unstable nature of the cyclic compounds or they may have been enzymatically degraded or integrated into the synthesis of the three linear lipids observed at the fourth day of fermentation. Their absence may also be as a result of the disappearance of the producing organisms. Ogueke & Aririatu (2004) reported that the production of an antibiotic “bacitracin” by *B. subtilis* (the predominant bacterium in the fermentation *P. macrophylla Benth* seed) inhibits the growth of other bacteria and causes the disappearance of *Micrococcus* sp. especially at 96 hr (fourth day) of fermentation. This may adversely affect the concentration of the volatile fermentation products of these organisms by resulting to an undetectable concentration or total absence as observed at the fourth day of this study.

The three volatile lipids observed at the fourth day of fermentation, have been reported to show different therapeutic properties. For example, the body weight regulating potentials of hexadecanoic acid attributed to its ability to control insulin secretion via central nervous system and suppression of the body's natural appetite‐suppressing signals from leptin and insulin in rat fed with a diet of 20% palmitic acid and 80% carbohydrate has been reported (Benoit et al., 2009). Though there are reports on the high metastasis‐inducing effect of hexadecanoic acid on human cancer (Pascual et al., 2016), the high concentration 9‐Octadecenoic acid (Z)‐, methyl ester in this fermented food may have a counter effect on the metastasis‐inducing potential of hexadecanoic acid. The consumption of the salt of 9‐Octadecenoic acid (Z)‐, methyl ester (oleate) has been reported to decrease breast cancer in women (Teres et al., 2008). This anti‐cancer effect of 9‐Octadecenoic acid (Z)‐, methyl ester (observed in higher concentration) may have a pronounced antagonistic effect on the metastasis‐inducing potential of hexadecanoic acid. As a monounsaturated omega‐9 fatty acid, 9‐Octadecenoic acid (Z)‐, methyl ester (oleic acid) consumption has been associated with an increased concentration of high‐density lipoprotein (HDL) and a concomitant decrease in low‐density lipoprotein (Martin‐Moreno et al., 1994). This indicates that fermented *P. macrophylla Benth* seed may be useful in the treatment and management of high blood pressure. A high percentage concentration of methyl seterate (methyl ester of octadecanoic acid) was also observed at the fourth day of fermentation. The bifunctional ability of the polar head group of this compound to attach metal cations and the solubility of its nonpolar chain in organic solvent makes it a notable surfactant and a softening agent. This may be responsible for the increase in softness and enhanced emulsification of fermented *P. macrophylla Benth* seed in palmitic oil as fermentation time increases.

## 5 CONCLUSION

Aside from softening the delicacy, a 4 day chance fermentation of cooked *P. macrophylla Benth* seed also reduces the eight component lipids present in the cooked unfermented seed to a more nutriceutical three component lipids (Hexadecanoic acid methyl ester, 9‐Octadecenoic acid (Z)‐methyl ester and Methyl stearate).

## CONFLICT OF INTEREST

None declared.
